# Predicting condensate formation of protein and RNA under various environmental conditions

**DOI:** 10.1186/s12859-024-05764-z

**Published:** 2024-04-02

**Authors:** Ka Yin Chin, Shoichi Ishida, Yukio Sasaki, Kei Terayama

**Affiliations:** 1 Graduate School of Medical Life Science, Yokohama City University, 1-7-29, Suehiro-cho, Tsurumi-ku, Yokohama, Kanagawa 230-0045 Japan; 2 RIKEN Center for Advanced Intelligence Project, 1-4-1, Nihonbashi, Chuo-ku, Tokyo, 103-0027 Japan; 3 MDX Research Center for Element Strategy, Tokyo Institute of Technology, 4259 Nagatsuta-cho, Midori-ku, Yokohama, Kanagawa 226-8501 Japan

**Keywords:** Liquid–liquid phase separation, Machine learning, Protein, RNA, Experimental conditions

## Abstract

**Background:**

Liquid–liquid phase separation (LLPS) by biomolecules plays a central role in various biological phenomena and has garnered significant attention. The behavior of LLPS is strongly influenced by the characteristics of RNAs and environmental factors such as pH and temperature, as well as the properties of proteins. Recently, several databases recording LLPS-related biomolecules have been established, and prediction models of LLPS-related phenomena have been explored using these databases. However, a prediction model that concurrently considers proteins, RNAs, and experimental conditions has not been developed due to the limited information available from individual experiments in public databases.

**Results:**

To address this challenge, we have constructed a new dataset, RNAPSEC, which serves each experiment as a data point. This dataset was accomplished by manually collecting data from public literature. Utilizing RNAPSEC, we developed two prediction models that consider a protein, RNA, and experimental conditions. The first model can predict the LLPS behavior of a protein and RNA under given experimental conditions. The second model can predict the required conditions for a given protein and RNA to undergo LLPS.

**Conclusions:**

RNAPSEC and these prediction models are expected to accelerate our understanding of the roles of proteins, RNAs, and environmental factors in LLPS.

**Supplementary Information:**

The online version contains supplementary material available at 10.1186/s12859-024-05764-z.

## Introduction

Liquid–liquid phase separation (LLPS) of biomolecules, such as proteins and RNAs, has attracted significant attention due to its central role in various cellular phenomena and implications in several diseases. LLPS is a physicochemical process that allows the formation and maintenance of condensates composed of specific biomolecules [1, 2]. These condensates show liquid-like properties that allow for fusion, exchange, and dissolution of surrounding components, and can respond to specific extracellular and intracellular signals [3]. Dysregulation of LLPS has been suggested to induce a phase transition from liquid-like condensates to solid-like condensates, leading to the formation of non-soluble aggregates [4, 5]. These non-soluble aggregates are characteristic features found in neurodegenerative diseases, including Alzheimer’s disease and amyotrophic lateral sclerosis [6, 7]. Given that LLPS is strongly associated with cellular phenomena and several diseases, it is important to evaluate the LLPS propensities of biomolecules to elucidate the relationship between their functions and diseases.

Recent studies have shown that the formation and maintenance of LLPS are regulated by RNA and environmental factors, such as surrounding pH and temperature, in addition to protein properties [8–10]. Numerous RNAs have been found within membraneless organelles, and it is suggested that various properties of RNAs, including their concentration, length, structure, and sequence, can influence the behavior of LLPS [10–15]. Additionally, several studies have reported that changes in environmental factors, such as pH, temperature, and ionic strength, can also alter the behavior of LLPS [9, 16, 17]. Therefore, proteins, RNAs, and environmental factors are important regulators of LLPS and should be considered together for a better understanding of LLPS as a biological phenomenon.

In recent years, there has been an increase in published LLPS-related databases, and the LLPS-related research utilizing machine learning (ML)-based models has intensified [18–27]. The previous models have mainly focused on identifying LLPS-related proteins, and their effectiveness has already been demonstrated. These models typically input a protein sequence or sequence-derived properties and output predictions regarding the behavior of LLPS [22–24]. Furthermore, a prediction model that takes a protein sequence and experimental conditions as input and output the propensity of the protein to undergo LLPS has recently been developed using LLPSDB, an LLPS-related database [18, 21]. These studies have identified several critical features of proteins that significantly influence LLPS behavior through analyses of the databases and assessments of the prediction models. However, prediction models that consider proteins, RNAs, and environmental factors have not been developed, despite their importance in LLPS regulation.

In this study, we aimed to develop prediction models that consider a protein, RNA, and experimental conditions. Since the performance of a prediction model is heavily influenced by the quality and quantity of a training dataset, having more detailed information is desirable. However, the experimental data corresponding to a single experiment is not available in the published databases. This is due to inconsistent recording formats, missing values, the use of range, and the use of multiple notations to record information from multiple experiments as a single data point. Thus, we first constructed a dataset, RNAPSEC (RNAPhaSep with detailed Experimental Conditions), with a single experiment as an entry by thoroughly reviewing the public literature referred to in RNAPhaSep [26] and manually collecting detailed experimental information from experiments involving a single protein and RNA. RNAPhaSep is a comprehensive database that contains biomolecules recorded in other LLPS-related databases, such as LLPSDB [18], PhaSePro [19], DrLLPS [20], and PhaSepDB [27]. Using RNAPSEC, we first developed a ML-based model to predict whether a given protein and RNA can induce LLPS under a given condition. This prediction model showed a performance of ROC-AUC 0.67. Moreover, the prediction model can be used to immediately predict the behavior of LLPS under various experimental conditions, thus allowing the construction of phase diagrams and providing insights into LLPS-related experiments. We also developed an ML-based model that predicted the experimental conditions required for a given protein and RNA to undergo LLPS. Notably, this study is an important step toward building a prediction model for LLPS that considers proteins, RNAs, and experimental conditions. RNAPSEC and codes for the prediction models are available in GitHub. The prediction models are also available in Google Colaboratory and are easily accessible to inexperienced programmers.

## Methods

**Fig. 1:**
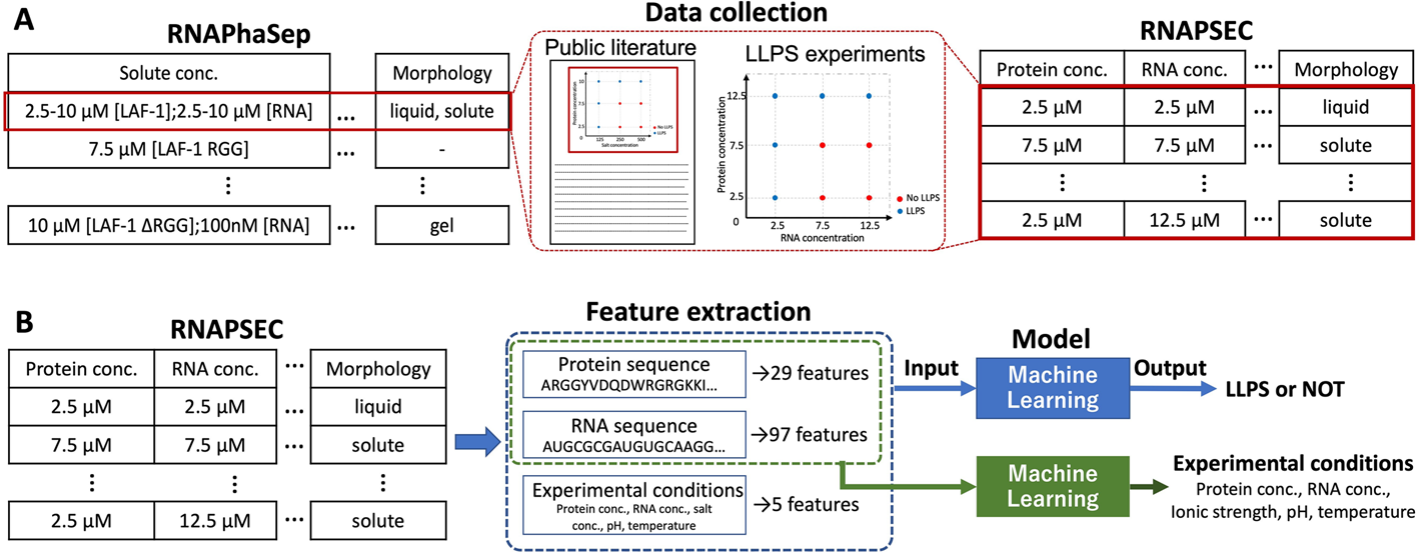
Overview of the construction process for RNAPSEC and prediction models. We first constructed RNAPSEC (**A**) and subsequently performed the model construction (**B**). **A** Overview of the dataset construction. The experimental information from RNAPhaSep, where multiple experiments were grouped together, was disassembled into individual experiments. To achieve this, we manually extracted the experimental information from the public literature stored in RNAPhaSep and recorded each experiment as one entry. **B** Overview of the model construction. We constructed two ML-based models using RNAPSEC. The first model predicts LLPS behavior using protein-derived features, RNA-derived features, and experimental conditions. The second model predicts the required conditions for a protein and RNA to undergo LLPS using protein-derived features and RNA-derived features. Abbreviations: conc., concentration

The overview of this study is illustrated in Fig. 1. We first constructed RNAPSEC (Fig. 1A) and then developed prediction models using a preprocessed dataset derived from RNAPSEC (Fig. 1B). RNAPSEC was constructed by manually collecting experimental information from the literature referred to in RNAPhaSep (“Construction of RNAPSEC” section). The preprocessed dataset was constructed by unifying the representation of protein sequences, RNA sequences, and experimental conditions and then transforming them into numeric features representing their properties (“Preprocessing of experimental conditions, protein sequences, and RNA sequences” section). To develop a model with useful outputs for LLPS-related experiments using the preprocessed dataset, two ML models were designed: one that predicts whether a given protein and RNA can undergo LLPS under a given experimental condition (“Training and validation method for the prediction model of LLPS behavior” section) and the other one that predicts the experimental conditions required for a given protein and RNA to undergo LLPS (“Training and validation method for the prediction model of experimental conditions to undergo LLPS” section).

### Construction of RNAPSEC

RNAPSEC was constructed by collecting experimental information from literature referenced in RNAPhaSep (Fig. 1A). Protein sequences, RNA sequences, protein concentration, RNA concentration, salt concentration, buffer pH, temperature, and condensate formation were manually recorded into RNAPSEC. The protein sequences were recorded in FASTA format, and the RNA sequences were recorded as single-letter sequences of nucleotides. Here, to construct the prediction models, experiments involving only a single protein and RNA were recorded. To simplify data processing, values and units were recorded in separate columns for protein, RNA, and salt concentrations. Additional parameters, including incubation time and other molecules, were recorded in the same manner as in RNAPhaSep. Links to original literature can be found in the “pmidlink” column of RNAPSEC. Similarly, detailed information on proteins and RNAs can be found in the corresponding columns of RNAPSEC. For example, the protein sequences, names, and Uniprot IDs have been listed in the columns of “protein_sequence”, “rnaphasep_protein_name”, and “rnaphasep_Uniprot ID”, respectively.

The results of LLPS-related experiments were recorded based on morphological characteristics of phase separation described in the literature, and four states were used: solute, liquid, gel, and solid. Solute represents non-LLPS, liquid represents the formation of liquid-like condensates, gel represents the formation of gel-like condensates, and solid represents the formation of solid-like condensates. When the results were not mentioned in the text, they were estimated from the size and shape of the condensates in the images. Images with spherical granules were classified as liquid-like condensates, images with reticular networks were classified as gel-like condensates, and images with isolated non-spherical objects were classified as solid-like condensates [1–5]. Data that could not be determined from the text or images were excluded.

### Preprocessing of experimental conditions, protein sequences, and RNA sequences

To develop prediction models, a preprocessed dataset was created by converting the protein and RNA sequences into numerical features and unifying the representation of experimental conditions over the dataset (Fig. 1B). We selected data points that were derived from LLPS-related experiments involving a single protein-RNA pair and had five experimental conditions: protein concentration, RNA concentration, salt concentration, pH, and temperature. Data involving small molecules or crowding agents were excluded.

The protein sequences were unified into single-letter amino acid sequences by removing the description on the first line of the FASTA format. Subsequently, 29 features, including amino acid composition, hydrophobicity, and isoelectric point, were calculated using the Protein Analysis module from the Biopython package [28]. From the RNA sequences, 97 features, including nucleotide composition and sequence periodicities, were calculated using the Mathfeature package [29]. Data points with protein or RNA sequence lengths less than 10 were excluded because the descriptor calculation tools did not support them. Detailed information about the features is provided in the Additional file 1: Tables S1 and S2.

The protein concentration, RNA concentration, pH, salt concentration, and temperature were used as experimental conditions. Measure units of the protein and RNA concentrations were unified as μM and were converted using a common logarithmic transformation. The salt concentration was converted to ionic strength using the pyEQL package [30]. Data points involving salts not supported by pyEQL were excluded. For the temperature, the units were unified to Celsius, and room temperature was defined as 25 degrees Celsius. No specific treatment was performed for the pH.

Finally, a total of 131 features, including 29 features from protein sequences, 97 features from RNA sequences, and 5 features from experimental conditions, were used for the model development. A dataset was also prepared in which the values of each feature were standardized to have a mean of 0 and a variance of 1.

### Training and validation method for the prediction model of LLPS behavior

The first model predicts whether a given protein and RNA can undergo LLPS under given experimental conditions using the 131 features converted from the protein, RNA, and experimental conditions (Fig. 1B). The prediction models were developed using seven different ML algorithms: Logistic Regression (LR), K-Nearest Neighbor (KNN), Support Vector Machine (SVM), Gaussian Naïve Bayes (GaussianNB), Random Forest (RF), Light Gradient Boosting Machine (LightGBM) [31], and Adaptive Boosting (AdaBoost) [32]. All algorithms except LightGBM were implemented using the scikit-learn library [33]. For LR, KNN, SVM, and GaussiaNB, the standardized dataset was used, and for RF, AdaBoost, and LightGBM, the non-standardized dataset was used.

The performances of the prediction models were assessed by repeated stratified group 10-fold cross-validation (SG10CV). In this study, each group corresponds to a dataset with the same protein sequence. In each fold, the dataset was split in a 9:1 ratio into a training and test dataset. The redundancy of the local sequences has not been considered in this process. The training dataset was used for hyperparameter tuning, and the prediction performance of the test dataset was evaluated using the trained model with the best hyperparameter and the training dataset. The hyperparameter was determined based on the highest ROC-AUC value using stratified five-fold cross-validation (CV). The search ranges for the hyperparameter tuning are shown in Additional file 1: Table S3. The SG10CV was repeated 10 times to perform a stable assessment. In each SG10CV, the average score of ROC-AUC was calculated, and the total average of the scores of the SG10CVs was used as the result of the repeated SG10CV. To analyze important features in predictions, the average feature importances for each SG10CV were calculated, and the average of the averaged feature importances was used as the result of the repeated SG10CV.

Phase diagrams were constructed by plotting the predicted results for shifting the protein and RNA concentrations while keeping the other features constant. The range of concentrations to be plotted in the phase diagram was determined for each protein-RNA pair. For each, the protein and RNA concentrations were divided into 20 points, from 1.0 less than the minimum value to 1.0 greater than the maximum value, and a total of 400 points were used for prediction. The prediction models were taken from the SG10CV that showed the highest ROC-AUC among the repeated SG10CV. Data involving the protein-RNA pair predicted in the phase diagram was included in the test data but not in the training data.

### Training and validation method for the prediction model of experimental conditions to undergo LLPS

The second model predicts the experimental conditions for a protein and RNA to undergo LLPS, based on the input of the features derived from the corresponding protein and RNA sequences (Fig. 1B). To predict multiple experimental conditions, classifier chains [34] were employed. The classifier chains are a sequence of classifiers connected in series, which can make multiple predictions by incorporating the prediction results of one previous classifier as input for the next classifier. The classifier chains model outputs experimental conditions in the following order: pH, temperature, protein concentration, RNA concentration, and ionic strength. Each experimental condition was classified into several classes according to its value and treated as a classification problem (Table 1). The pH was classified into three classes: acidic, neutral, and basic; the temperature into three classes: low temperature, room temperature, and high temperature; the protein concentration, RNA concentration, and ionic strength into five classes according to 20%, 40%, 60%, 80%, and 100% of the value distribution. The classifier chains were implemented using the scikit-learn library [33]. Due to the unbalanced amount of data in each class, for each experimental condition, a macro-averaged ROC curve was built from the total predicted results of test datasets in group 10-fold CV (G10CV) and evaluated using its ROC-AUC. In G10CV, each group corresponds to a dataset with the same protein sequence.

**Table 1:** Classification of experimental values into classes. Each experimental condition was classified into three or five classes depending on its value

Class	1	2	3	4	5
pH	0 to 7.0	7.0 to 8.0	8.0 to 14	–	–
Temp. (°C)	0 to 25	25 to 30	30 to 40	–	–
Ionic strength	0 to 0.032	0.032 to 0.066	0.066 to 0.14	0.14 to 0.17	0.17 to 0.40
Protein conc. (Log μM)	−1.64 to −0.49	−0.49 to −0.29	−0.29 to 0.84	0.84 to 1.5	1.5 to 2.7
RNA conc. (Log μM)	−4.7 to −3.7	−3.7 to −2.4	−2.4 to −1.0	−1.0 to −0.12	−0.12 to 2.3

## Results and discussion

### Data contents in RNAPSEC

**Fig. 2:**
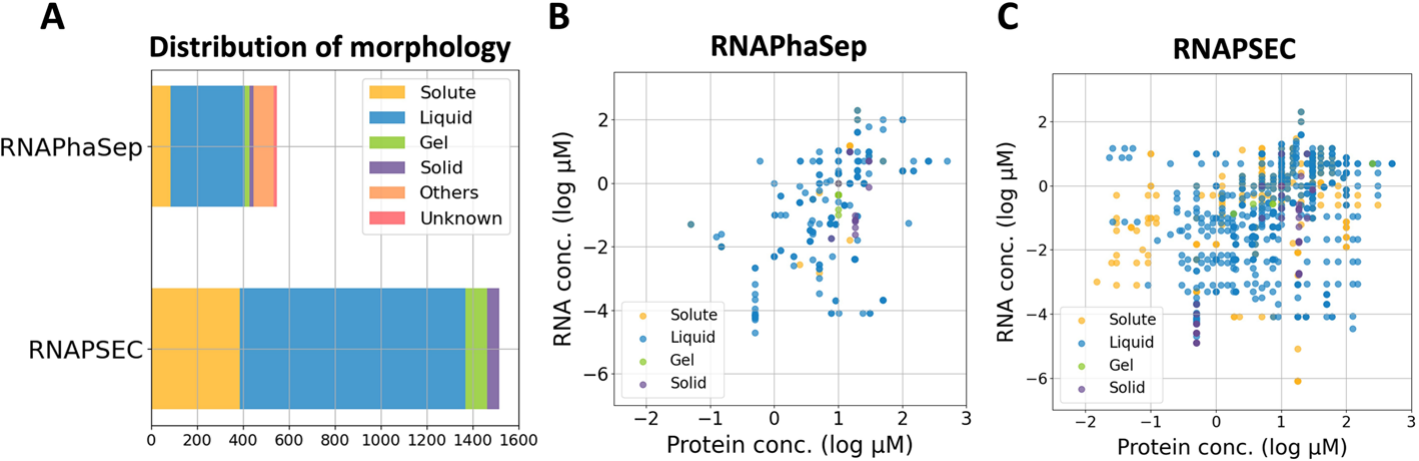
Distribution of morphology for phase separation and protein-RNA concentrations in RNAPShaSep and RNAPSEC. **A** The distribution of phase separation morphology in the experimental data consisted of a single protein and RNA in RNAPSEC and RNAPhaSep. Others represent data where more than one morphology has been recorded, and unknown represents data where no morphology has been recorded. **B** The distribution of protein and RNA concentrations in preprocessed RNAPhaSep. **C** The distribution of protein and RNA concentrations in preprocessed RNAPSEC. In **B** and **C**, the orange dots show data where LLPS did not occur, the blue dots show data where liquid-like condensates were formed, the green dots show data where gel-like condensates were formed, and the purple dots show data where solid-like condensates were formed

Figure 2A shows the distribution of morphologies for RNAPSEC and RNAPhaSep. As a result, both the total amount and the amount in each form were increased in RNAPSEC compared to RNAPhaSep under the same filter conditions. Currently, RNAPSEC contains a total of 1514 data points, including 385 solute data points without LLPS, 984 liquid data points with liquid-like condensates, 92 gel data points with gel-like condensates, and 53 solid data points with solid-like condensates. These experiments consisted of 37 proteins, including severe acute respiratory syndrome coronavirus 2 (SARS-CoV-2) nucleoproteins, fused in sarcoma (FUS) proteins, and TAR DNA-binding protein 43 kDa (TDP- 43) (Additional file 1: Figure S1A), with 96 unique sequences and 147 RNAs. Among the 1514 data points, 40 data points consisting of 13 protein sequences are negative data that do not cause LLPS under any conditions in the recorded range. Also, RNAPSEC contains several protein sequences with sequence deletions or amino acid substitutions, resulting in multiple variants for a single protein (Additional file 1: Figure S1B). RNAPhaSep (Component type = “RNA + Protein”) contains 86 solute data points, 323 liquid data points, 21 gel data points, 18 solid data points, 14 data points where experimental results are not described, and 87 data points with multiple descriptions (labeled as “Unknown” and “Other” in Fig. 2A). RNAPSEC was designed to avoid the inclusion of ambiguous entries, such as missing or multiple descriptions of experimental results. Note that the number of proteins and RNAs included in RNAPSEC appears to be small compared to other LLPS-related databases because only data that have all RNAs, proteins, and experimental conditions were collected.

Figure 2B and C shows the distribution of protein and RNA concentrations in RNAPhaSep and RNAPSEC, respectively. The protein and RNA concentrations in RNAPhaSep were preprocessed in the same way as in RNAPSEC. As the protein and RNA concentrations were recorded in a single column in RNAPhaSep, data with the following descriptions were excluded: data where it was unclear whether the recorded concentration referred to a protein or an RNA, where either concentration was not recorded, or where multiple experimental results were described. When a range notation or multiple values were mentioned, the average was used as the corresponding value. To compare the number of experiments, both datasets include data with small molecules or crowding agents. As a result, there are noticeable differences in the distribution of protein and RNA concentrations between RNAPhaSep and RNAPSEC. In the case of RNAPhaSep, the distribution appears scattered, suggesting that a large amount of information may be missing (Fig. 2B). In contrast, RNAPSEC showed a broad distribution of protein and RNA concentrations (Fig. 2C). In addition, the minimum and maximum values of the protein and RNA concentrations in RNAPSEC were more extended than those in RNAPhaSep in almost all morphologies (Additional file 1: Table S4). Therefore, RNAPSEC has a potential to provide a wide range of information regarding LLPS-related experiments, in addition to offering a greater quantity of data.

Previous studies have shown that changes in protein and RNA concentrations can alter the behavior of LLPS [9], suggesting a possible trend between concentration changes and LLPS behavior. However, no obvious tendencies were identified from the distribution of protein and RNA concentrations in RNAPSEC (Fig. 2C). This result suggests that LLPS is a complex phenomenon regulated by multiple experimental parameters and properties of biomolecules. It is also likely that the lack of trends from the distribution is due to the limited number of data points.

### Evaluation results of the model in predicting the LLPS behavior of a protein and RNA under given conditions

**Fig. 3:**
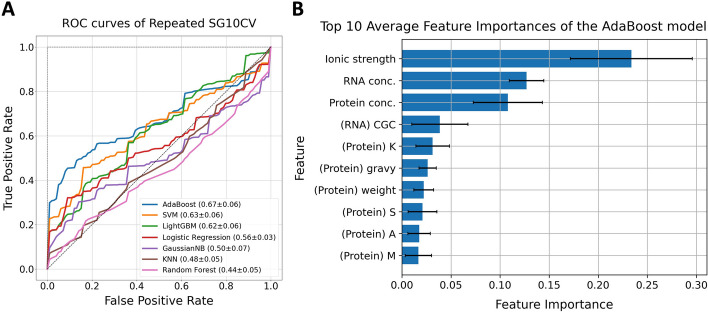
ROC curves and feature importances of ML models in predicting LLPS behavior. **A** The LR, KNN, SVM, GaussianNB, RF, LightGBM, and AdaBoost models were evaluated using repeated SG10CV, and their performances were assessed using ROC curves. The average curve for each SG10CV iteration of the repeated SG10CV was calculated, and the final result is shown as the total average curve. The values in brackets represent the overall average ROC-AUC values. **B** The top 10 average feature importances of the repeated SG10CV for the AdaBoost model are shown in the figure. The average value was calculated for each model trained within each SG10CV, and the average across all SG10CVs was calculated for the final result. Error bars represent standard deviations within each SG10CV

Evaluation results of the models predicting LLPS behavior are shown in Fig. 3A. The model predicting the LLPS behavior was trained and evaluated using 851 data recorded in RNAPSEC, including 294 solute data as negative data and 557 liquid data as positive data. These 851 data were selected from RNAPSEC by filtering the 1514 data points for the following conditions: solute or liquid data point; no missing value in the protein concentration, RNA concentration, salt concentration, temperature, and pH of solution; and no crowding agents or small molecules. The prediction model takes input features derived from a protein, RNA, and experimental conditions. It then outputs a prediction on whether the protein-RNA pair can undergo LLPS under the experimental conditions. We compared the performances of the prediction models using seven different algorithms, LR, KNN, SVM, GaussianNB, RF, LightGBM, and AdaBoost. The performance of each prediction model was estimated by repeated SG10CV using a total of 72 protein sequences as group labels. As a result, the AdaBoost model, the SVM model, and the LightGBM model showed superior performances, with the AdaBoost model showing the highest ROC-AUC of 0.67 (Fig. 3A). Recent studies have reported that peptide compositions of LLPS-related proteins are distinctive from the proteome and that Intrinsically Disordered Regions (IDRs) could provide the driving force for LLPS [2, 5, 22]. To examine the influence of such local sequences in the LLPS behavior, we have also developed four similar models that considered di/tri-peptide compositions in the protein sequences and the sequential features in the IDRs, respectively, as shown in Additional file 1: Figure S2. The results showed that the ROC-AUCs of the AdaBoost-based models were 0.62 and 0.63, which were lower than those of the original model. Furthermore, to confirm the effects of the features in the prediction, feature importances of the model that performed the best in the repeated SG10CV were calculated (Fig. 3B). As a result, ionic strength, protein concentration, and RNA concentration were significantly more important than the other features. In fact, the models based only on the experimental conditions can predict LLPS behavior to some extent (Additional file 1: Figure S3). This suggests that the prediction results were heavily influenced by these experimental conditions.

As shown in Fig. 3A, the maximum ROC-AUC obtained in this study was 0.67, indicating that the models did not perform better compared to previous studies that predicted LLPS behavior based on a protein sequence alone. For instance, the performances mentioned in the respective papers for PSAP [22] and PSPredictor [24] were an ROC-AUC of 0.88 and an accuracy of 94.71%, respectively. The performance of the proposed model could be attributed to the complexity of our prediction model, which considers three different factors: a protein, RNA, and experimental conditions. Such added complexity renders the prediction more challenging compared to considering a protein alone. However, given the intricate nature of LLPS behavior—a phenomenon influenced by the interplay of these three factors—prediction models should account for all these parameters.

From another perspective, the model performances might have been influenced by the composition of the training dataset. RNAPSEC was constructed by expanding the experimental information for each data of RNAPhaSep; therefore, many data with the same sequence were included. In general, it is difficult to predict different results from similar inputs, and this may lead to a decrease in model performance. For the same reason, the importance of the features derived from sequences with small differences in the dataset may have decreased, while the importance of experimental conditions with large differences among the dataset may have increased. To ensure the reliability of the prediction model, it is desirable to train on the values used in experiments. It is therefore desirable to expand RNAPSEC with unique sequences and experimental conditions to develop high-performance models and enable more detailed analyses.

### Construction of phase diagrams using the prediction model provides insight into LLPS-related experiments

**Fig. 4:**
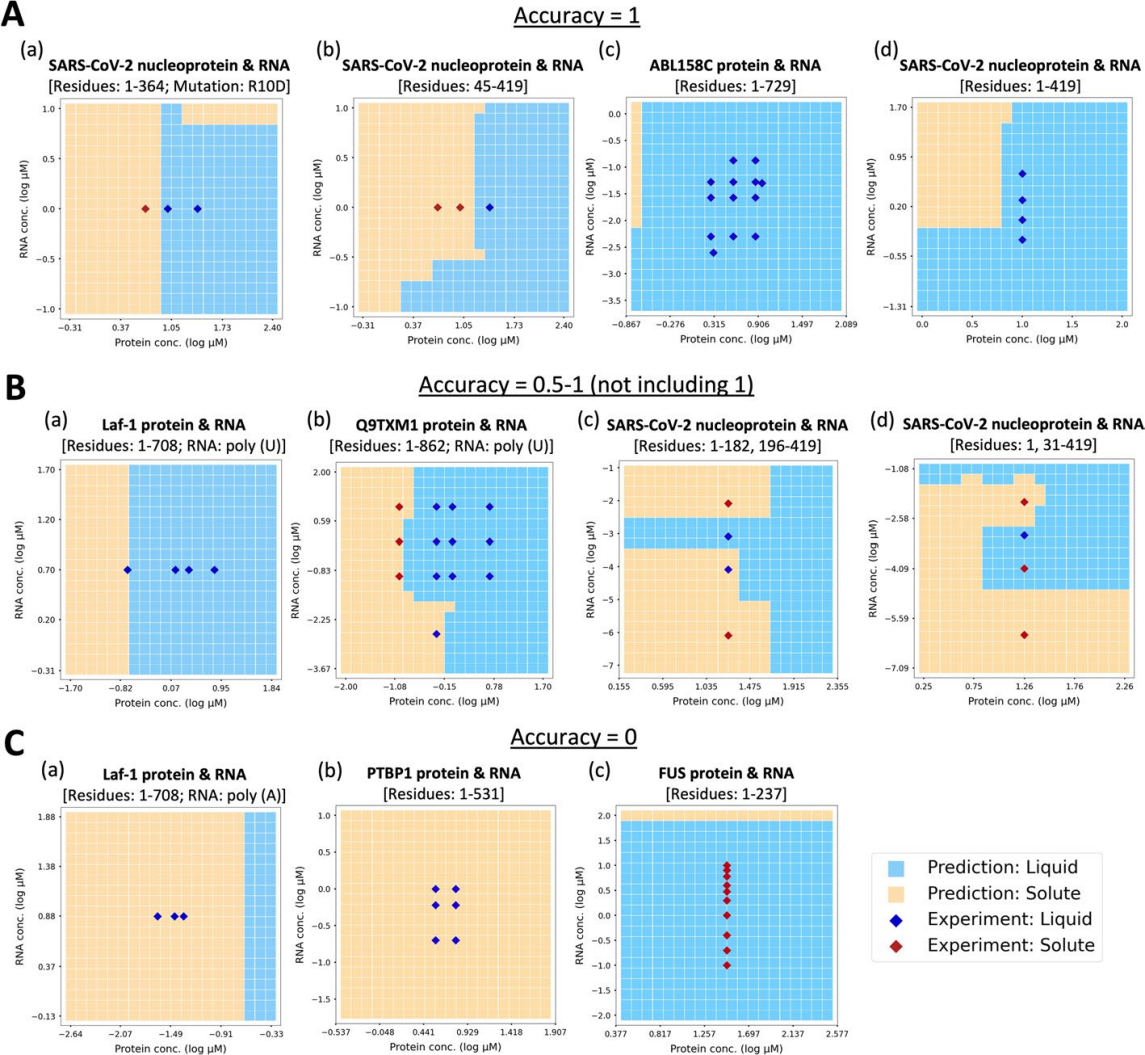
Examples of phase diagrams constructed by the results of the prediction model. Phase diagrams were generated from the prediction results of samples in which the protein and RNA concentrations were shifted at regular intervals. **A** Examples of phase diagrams where the accuracy score calculated from the predictions of the corresponding experimental data was 1. **B** Examples of phase diagrams where the accuracy score calculated from the predictions of the corresponding experimental data was between 0.5 and 1 (not including 1). **C** Examples of phase diagrams where the accuracy score calculated from the predictions of the corresponding experimental data was 0. In each phase diagram, the orange squares represent the samples predicted as non-LLPS, the light blue squares represent the samples predicted to form liquid-like condensates, the red rhombuses represent the samples where no LLPS occurred in experiments, and the blue rhombuses represent the samples where liquid-like condensates were formed in experiments

The prediction model developed above can immediately predict the LLPS behavior and is useful for the rapid construction of a phase diagram showing the LLPS behaviors under various conditions. In Fig. 4, predicted phase diagrams were displayed separately according to the accuracy score calculated from the prediction results of experimental data recorded in RNAPSEC for each protein-RNA pair. Figure 4A represents examples where the accuracy was 1.0, Fig. 4B represents examples where the accuracy was 0.5–1.0, and Fig. 4C represents examples where the accuracy was 0. In Fig. 4A, the orange squares were more common in areas with the red rhombuses, and the light blue squares in areas with the blue rhombuses. Intriguingly, in experiments where only liquid-like condensates were observed, the LLPS behaviors were predicted to be altered by shifting the input values of protein concentration and RNA concentration (Fig. 4A c, d). Under these conditions, the actual LLPS behavior may show similar changes to the prediction results. Similar to Fig. 4A, the boundary of the prediction results in Fig. 4B almost aligns with the experimental results. This suggests that it may be possible to construct a reliable phase diagram, even with an accuracy of less than 1. Moreover, RNAPSEC contains a large amount of data regarding the severe acute respiratory syndrome coronavirus 2 (SARS-CoV-2) nucleoprotein, and many of these phase diagrams are appropriate (Additional file 1: Figure S4A). Furthermore, in the examples where the behavior of LLPS changed with shifting the RNA concentrations, similar predictions were obtained (Fig. 4B b-d). This result shows that the prediction model can predict the LLPS behaviors considering the influence of RNA concentration. Therefore, our model, which was trained on the data consisting of individual experiments, has the potential to predict LLPS behavior under a wide variety of experimental conditions.

However, as shown in Figs. 4C and Additional file 1: S4B, there were some cases in which the prediction results deviated significantly from the experimental results. This indicates that it is difficult to achieve completely accurate predictions for all scenarios with the current model. Future efforts to expand the number of unique biomolecules and the amount of data included in RNAPSEC are expected to improve the performance of the model and enable its application to the comprehensive screening of LLPS conditions.

### Evaluation result of the model in predicting experimental conditions for a protein and RNA to undergo LLPS

**Fig. 5:**
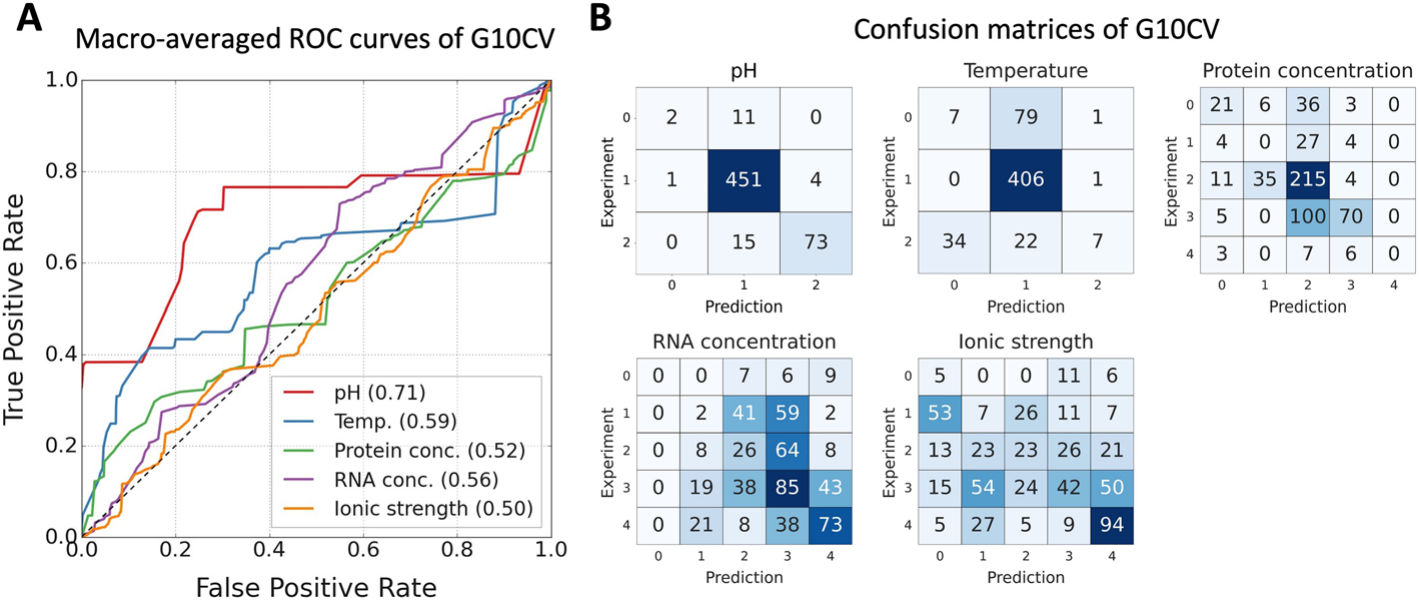
Performance of the classifier chains model with the AdaBoost models in predicting five experimental conditions. **A** The model performance was evaluated using the G10CV and the results are shown in the macro-averaged ROC curves. Each curve shows the result of the model predicting the experimental condition for the corresponding color in the legend. Values in the brackets represent the macro-average of the ROC-AUCs for each class shown in Table 1. **B** Confusion matrices built from the predictions of the G10CV. Axis labels correspond to the classes in Table 1

Furthermore, we developed a model that predicts the required experimental conditions to undergo LLPS using the classifier chains model based on AdaBoost, which showed the best performance in the previous prediction task. Each AdaBoost model was trained with the default hyperparameters and evaluated using 557 liquid data from RNAPSEC. The model takes the input features derived from protein and RNA sequences and outputs pH, temperature, protein concentration, RNA concentration, and ionic strength. The performance of the prediction model was evaluated by G10CV using protein sequences were treated as group labels. The macro-averaged value of the ROC-AUCs for pH was above 0.71 (Fig. 5A) and showed relatively better performance in predicting each class (Fig. 5B). However, for other factors, each ROC-AUC was around 0.50 and showed that the predictions accuracy of each class was close to random chance. Therefore, it is difficult for the model to accurately predict the experimental conditions based on the current dataset.

## Conclusion

In this study, we developed two prediction models that consider three elements: protein, RNA, and experimental conditions. Our study represents an initial step toward developing an LLPS prediction model that considers all these factors. To achieve this, we first constructed a dataset called RNAPSEC, which comprises 1514 data points about to LLPS-related experiments involving a single protein and RNA. Within RNAPSEC, each individual experiment was recorded as a single data point, providing information on LLPS-related experiments under various conditions. Using RNAPSEC, we developed two different models related to LLPS. The first model was able to predict the LLPS behavior of a given protein and RNA under given experimental conditions, which had not been considered in previous studies, with an ROC-AUC of 0.67. This model allows large-scale screening and the construction of phase diagrams, which are expected to be useful in planning experiments. The second model can predict the experimental conditions under which a given protein and RNA will undergo LLPS. This is the first model to output the experimental conditions required for LLPS. The ROC-AUCs of the model were 0.50, 0.52, 0.56, 0.59, and 0.71 for the five experimental conditions, ionic strength, protein concentration, RNA concentration, temperature, and pH, respectively. Although further improvements in prediction accuracy and applicability domain are required, these models are highly reliable in predicting experimental conditions because they were trained on individual experimental data. Expanding the scope of RNAPSEC and enhancing model performance will enable more detailed analysis and prediction of the complex relationships among proteins, RNA, and environmental factors in LLPS. This will contribute to a further understanding of LLPS by uncovering the complex interplay among these three factors.

### Supplementary Information

**Additional file 1: **Tables S1–S4 and Figures S1–S4.

## Data Availability

For RNAPSEC, the prediction models, and the Google Colaboratory version of the prediction models are available at GitHub repository (https://github.com/ycu-iil/RNAPSEC).

## Abbreviations

LLPS: Liquid–liquid phase separation
ML: Machine learning
ROC: Receiver operating characteristic
AUC: Area under curve
LR: Logistic regression
KNN: K-nearest neighbor
SVM: Support vector machine
GaussianNB: Gaussian Naïve Bayes
RF: Random forest
LightGBM: Light gradient boosting machine
AdaBoost: Adaptive boosting
SG10CV: Stratified group 10-fold cross-validation
CV: Cross-validation
G10CV: Group 10-fold cross-validation
IDR: Intrinsically disordered region
