# Supplementary material for: Predicting condensate formation of protein and RNA under various environmental conditions
Source: BMC Bioinformatics. 2024 Apr 2;25:143. doi: 10.1186/s12859-024-05764-z (PMC10988968; doi:10.1186/s12859-024-05764-z)
Supplement: Supplementary file 1 — Additional file 1: Tables S1–S4 and Figures S1–S4. [file 12859_2024_5764_MOESM1_ESM.docx]

Supporting Information

Predicting condensate formation of protein and RNA under various environmental conditions

Ka Yin Chin^1^, Shoichi Ishida^1^, Yukio Sasaki^1^ and Kei Terayama^1,2,3*^

^1^ Graduate School of Medical Life Science, Yokohama City University, 1-7-29, Suehiro-cho, Tsurumi-ku, Kanagawa 230-0045, Japan

^2^ RIKEN Center for Advanced Intelligence Project, 1-4-1, Nihonbashi, Chuo-ku, Tokyo 103-0027, Japan.

^3^ MDX Research Center for Element Strategy, Tokyo Institute of Technology, 4259 Nagatsuta-cho, Midori-ku, Yokohama, Kanagawa, 226-8501, Japan.

* To whom correspondence should be addressed.

Contact: terayama@yokohama-cu.ac.jp

**Contents:**

Table S1. Feature extraction from a protein sequence.

Table S2. Features extraction from an RNA sequence.

Table S3. Hyper-parameter tuning for the models to predict the LLPS behavior.

Table S4. Minimum and maximum concentrations of proteins and RNA in RNAPhaSep and RNAPSEC

Figure S1. The detailed information about protein and protein sequences in RNAPSEC.

Figure S2. ROC curves for the prediction models with additional features of di/tri-peptide composition and IDR-derived features.

Figure S3. ROC curves for the models predicting LLPS behavior based only on the experimental conditions.

Figure S4. Examples of phase diagrams constructed by the results of the prediction model.

**Table S1. Feature extraction from a protein sequence.** The features described in Table S1 were extracted or calculated from a protein sequence using Biopython [1]. The following description is based on the document of Biopython [2].

| Feature | Detail | Dimension |
| --- | --- | --- |
| Amino acid composition | The composition of each amino acid.   - A, C, E, D, G, F, I, H, K, M, L, N, Q, P, S, R, T, W, V, Y | 20 |
| Molecular weight | The molecular weight of a protein. | 1 |
| Gravy | Calculated the hydrophobicity of a protein [3]. | 1 |
| Aromaticity | The relative frequency of F, W, Y. | 1 |
| Instability | Calculated the stability of protein using the method of Guruprasad *et al.* [4]. | 1 |
| Flexibility | Calculated the average flexibility of a full-length protein using the method of Vihinen *et al*. [5]. | 1 |
| Isoelectric point | Return the isoelectric point of a protein. | 1 |
| Secondary structure fraction | Return the fraction of amino acids below.  - Amino acids in helix: V, I, Y, F, W, L - Amino acids in turn: N, P, G, S - Amino acids in sheet: E, M, A, L | 3 |

**Table S2. Feature extraction from an RNA sequence.** Previous studies have suggested that structural and sequential features of RNA could be relevant to the regulation of phase separations [6, 7]. Therefore, we extracted the descriptors listed in the table from the RNA sequences using MathFeature [8] and used them as inputs for the model. The following description is based on the description in the text of MathFeature.

| Feature groups | Feature | Dimension |
| --- | --- | --- |
| Nucleic acid composition | Nucleic Acid composition   - A, G, U, C | 4 |
|  | Dinucleotide composition   - AA, AG, AU, AC, GG, GA, GU, GC, UU, UA, UG, UC, CC, CA, CG, CU | 16 |
|  | Trinucleotide composition   - AAA, AGA, AUA, ACA, GGA, GAA, GUA, GCA, UUA, UAA, UGA, UCA, CCA, CAA, CGA, CUA, AAG, AGG, AUG, ACG, GGG, GAG, GUG, GCG, UUG, UAG, UGG, UCG, CCG, CAG, CGG, CUG, AAU, AGU, AUU, ACU, GGU, GAU, GUU, GCU, UUU, UAU, UGU, UCU, CCU, CAU, CGU, CUU, AAC, AGC, AUC, ACC, GGC, GAC, GUC, GCC, UUC, UAC, UGC, UCC, CCC, CAC, CGC, CUC | 64 |
| Entropy | Shannon entropy for each k-mer (k = 1, 2) | 2 |
|  | Tsallis entropy for each k-mer (k = 1, 2) | 2 |
| Fourier transform | Z-curve + Fourier  The average and peak values were used | 2 |
|  | Real + Fourier  The average and peak values were used | 2 |
|  | Binary + Fourier  The average and peak values were used | 2 |
| Open reading frame (ORF) | The average length of ORF was used | 1 |
| Fickett score | Fickett score calculated from a full-length and ORF were used | 2 |

**Table S3. The ranges of hyper-parameters for the models to predict the LLPS behavior.** Hyper-parameters and their search ranges for each model are listed. The numbers listed in the search ranges indicate the minimum and maximum values of the searching range. The hyperparameters containing “None" in the search ranges were searched for “None" in addition to the number between the minimum and maximum values. The hyperparameters for each model were tuned with 100 search trials using Optuna [9].

| Model | Hyperparameter | Types | Search ranges |
| --- | --- | --- | --- |
| LightGBM | lambda l1 | Real numbers | [1.00×10^-8^, 10] |
|  | lambda l2 | Real numbers | [1.00×10^-8^, 10] |
|  | The maximum number of leaves | Integers | [2, 256] |
|  | Feature fraction | Real numbers | [0.4, 1] |
|  | Bagging fraction | Real numbers | [0.4, 1] |
|  | Bagging frequency | Integers | [1, 7] |
|  | Min child samples | Integers | [5, 100] |
|  | Random state | Integers | 1, 10, 20, …, 100 |
| RF | Number of estimators | Integers | 10, 100, 200, …, 500 |
|  | Criterion | Categorical numbers | Gini, entropy |
|  | Max depth | Integers | 1, 2, 3, 4 |
|  | Max feature | Categorical numbers | Sqrt, log2, None |
| AdaBoost | Max depth of estimators in the Decision tree as base estimator. | Integers | 5, 6, 7, …, 10, None |
|  | Learning rate | Real numbers | 0.5, 1.0, 1.5, 2.0, 2.5 |
| GaussianNB | Variance smoothing | Real numbers | [-log 9, 1] |
| LR | C | Real numbers | [1.00×10^-5^, 1.00×10^6^] |
|  | Random state | Integers | 0, 20, 40, …, 100 |
| KNN | Number of neighbors | Integers | 1, 3, 5, …, 21 |
|  | Weights | Categorical numbers | Uniform, distance |
|  | Power parameter for the Minkowski metric | Integers | 1, 2 |
| SVM | C | Real numbers | [1.00×10^-10^, 1.00×10^6^] |
|  | Gamma | Real numbers | [1.00×10^-10^, 1.00×10^6^] |

**Table S4. Minimum and maximum concentrations of proteins and RNA in RNAPhaSep and RNAPSEC**. This table presents the minimum and maximum concentrations of proteins and RNA for each morphology in RNAPhaSep and RNAPSEC, corresponding to Figures 2B and C.

|  | Minimum (Log (μM)) | | Maximum (Log (μM)) | |
| --- | --- | --- | --- | --- |
|  | RNAPhaSep | RNAPSEC | RNAPhaSep | RNAPSEC |
| Solute (Protein conc.) | 1.00 | 0.27 | 1.00 | 2.40 |
| Liquid (Protein conc.) | -1.30 | -1.64 | 2.41 | 2.70 |
| Gel (Protein conc.) | 0.89 | -0.30 | 1.45 | 1.48 |
| Solid (Protein conc.) | -1.30 | -1.82 | 2.00 | 2.48 |
| Solute (RNA conc.) | -0.99 | -0.87 | -0.37 | 0.70 |
| Liquid (RNA conc.) | -4.71 | -4.71 | 2.30 | 2.30 |
| Gel (RNA conc.) | -1.74 | -4.90 | 1.00 | 1.00 |
| Solid (RNA conc.) | -2.82 | -6.10 | 2.30 | 2.30 |

 **Fig. S1. The detailed information about protein and protein sequences in RNAPSEC.** (A) The quantity of data for each protein in RNASEC. (B) The number of unique sequences per protein is shown in the figure. In both, Classifications of proteins are according to the RNAPhaSep criteria. Abbreviations: SARS2 N protein, SARS-CoV-2 nucleocapsid protein.


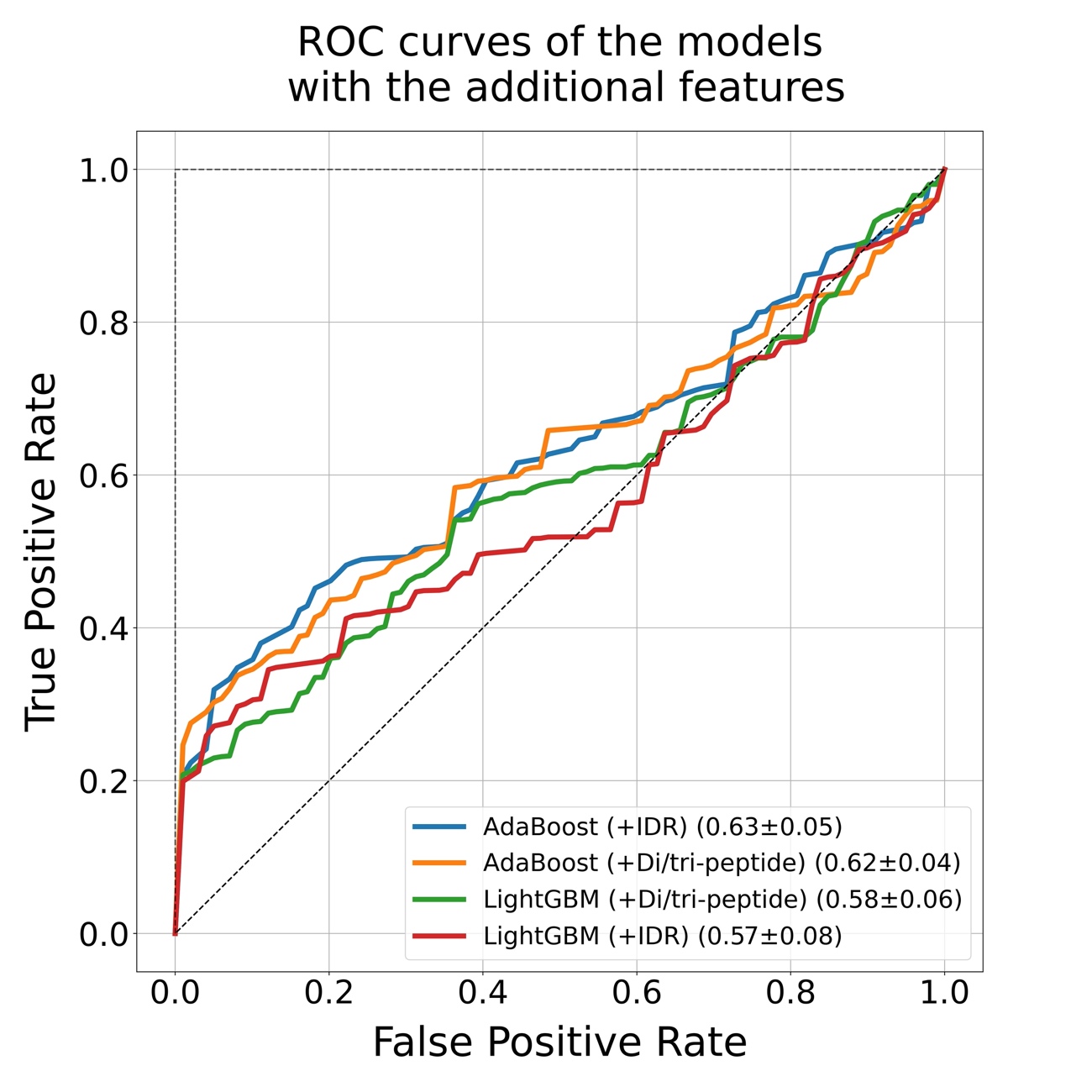


**Fig. S2. ROC curves for the prediction models with additional features of di/tri-peptide composition and IDR-derived features.** We have also developed two models predicting LLPS behaviors, one with di/tri-peptide compositions and one with IDR-derived features added to the input features of the original model, respectively. The di/tri-peptide compositions were calculated using Mathfeature [8] and consisted of the 8400 features. The IDRs were predicted from MobiDB-lite [10], one of the powerful IDR predictors. The IDR-derived features consisted of a total of 430 features, including the 29 features of Table S1 calculated from the IDRs, the percentage of IDR content, and the 400 features of the IDR dipeptide composition. For data where no IDRs were predicted in the full-length sequence, the IDR-derived features were complemented with 0. Using these input features, the AdaBoost and LightGBM-based models were developed respectively and evaluated by repeated stratified group 10-fold cross-validation using protein sequences as group labels, the same as in the original model. The performances of the models are shown in the ROC curves.


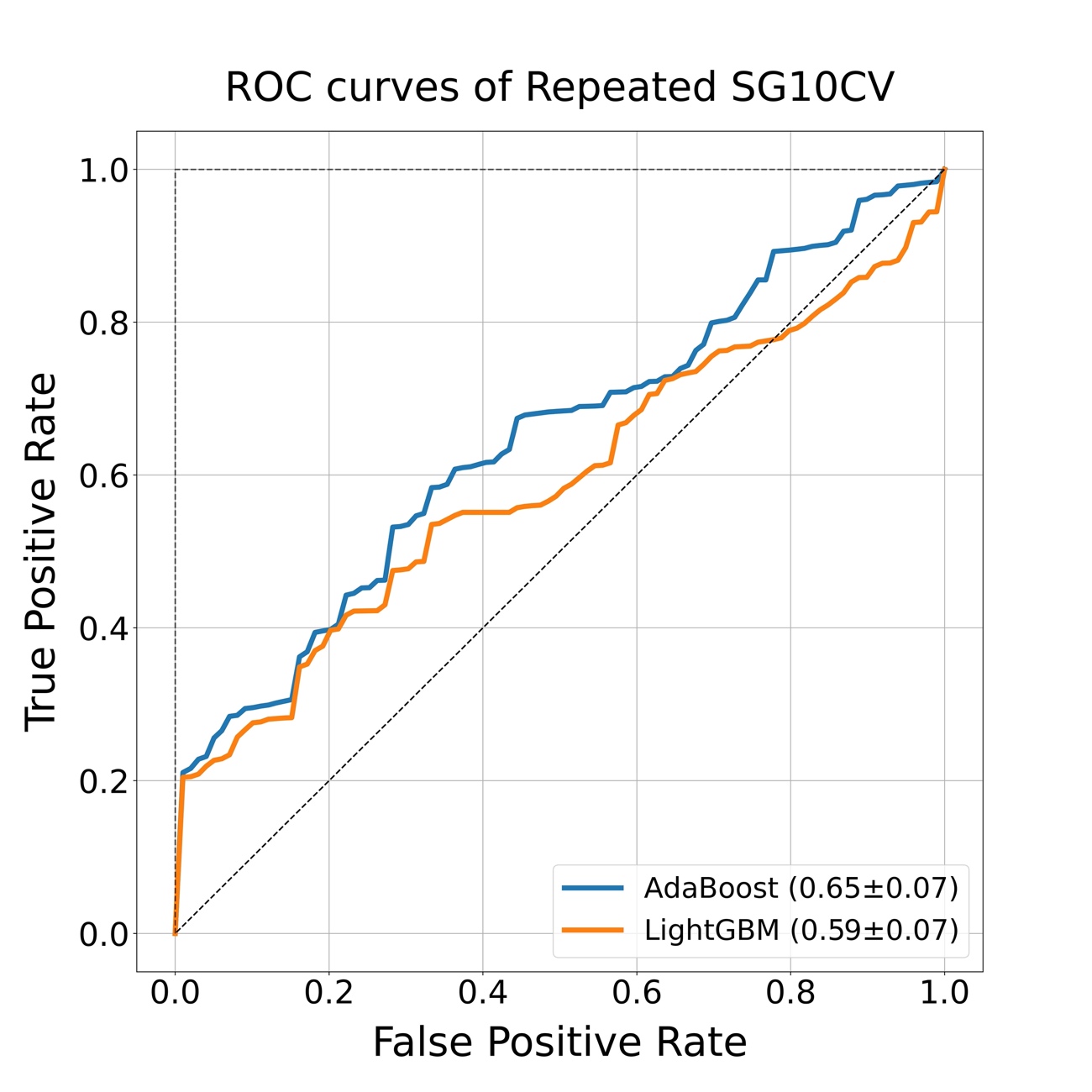


**Fig. S3. ROC curves for the models predicting LLPS behavior based only on the experimental conditions**. We developed AdaBoost and LightGBM-based models to predict the LLPS behavior from the inputs of the experimental conditions, ionic strength, protein concentration, RNA concentration, temperature, and pH. The performances of the models were assessed with repeated stratified 10-fold cross-validations and the results are shown in the ROC curves.


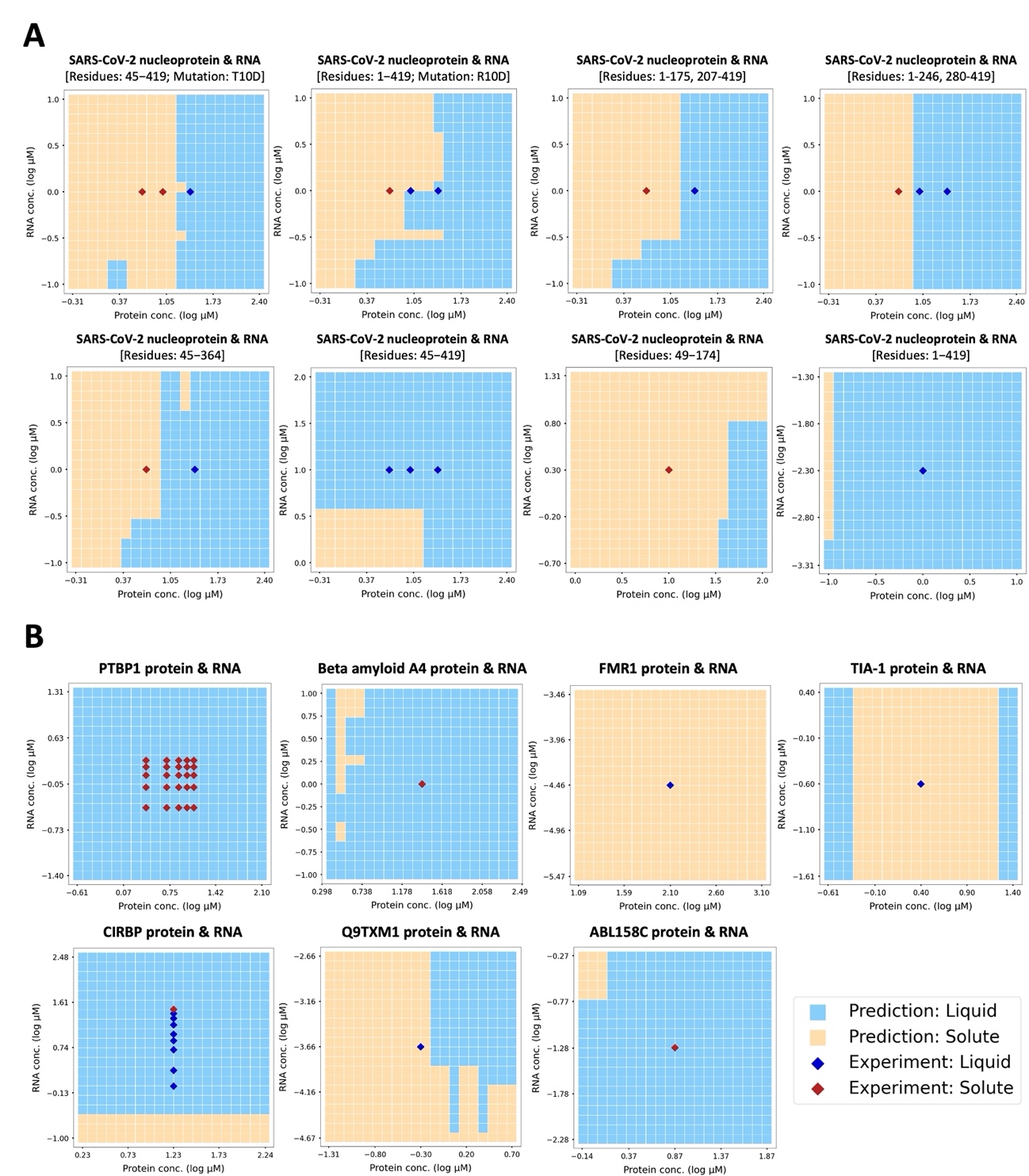


**Fig. S4. Examples of phase diagrams constructed by the results of the prediction model.** (A) Examples of phase diagrams using the SARS-CoV-2 nucleoprotein and RNA. The description in each square bracket indicates the characteristics of each protein sequence. (B) Failed examples of predicted phase diagrams. In each phase diagram, the orange squares represent the samples predicted as non-LLPS, the light blue squares represent the samples predicted to form liquid-like condensates, the red rhombuses represent the samples where no LLPS occurred in experiments, and the blue rhombuses represent the samples where liquid-like condensates were formed in experiments.

References

[1] P. J. A. Cock *et al.*, “Biopython: freely available Python tools for computational molecular biology and bioinformatics,” *Bioinformatics*, vol. 25, no. 11, pp. 1422–1423, Jun. 2009, doi: 10.1093/bioinformatics/btp163.

[2] “Analyzing protein sequences with the ProtParam module,” Biopython.org. <https://biopython.org/wiki/ProtParam> (accessed: Sep. 4,2023).

[3] J. Kyte and R. F. Doolittle, “A simple method for displaying the hydropathic character of a protein,” *J. Mol. Biol.*, vol. 157, no. 1, pp. 105–132, May 1982, doi: 10.1016/0022-2836(82)90515-0.

[4] K. Guruprasad, B. V. B. Reddy, and M. W. Pandit, “Correlation between stability of a protein and its dipeptide composition: a novel approach for predicting in vivo stability of a protein from its primary sequence,” *Protein Eng.*, vol. 4, no. 2, pp. 155–161, Jan. 1990, doi: 10.1093/protein/4.2.155.

[5] M. Vihinen, E. Torkkila, and P. Riikonen, “Accuracy of protein flexibility predictions,” *Proteins*, vol. 19, no. 2, pp. 141–149, Jun. 1994, doi: 10.1002/prot.340190207.

[6] C. Roden and A. S. Gladfelter, “RNA contributions to the form and function of biomolecular condensates,” *Nat. Rev. Mol. Cell Biol.*, vol. 22, no. 3, pp. 183–195, Mar. 2021, doi: 10.1038/s41580-020-0264-6.

[7] M. Garcia-Jove Navarro *et al*., “RNA is a critical element for the sizing and the composition of phase-separated RNA–protein condensates,” *Nat. Commun.*, vol. 10, no. 1, p. 3230, Jul. 2019, doi: 10.1038/s41467-019-11241-6.

[8] R. P. Bonidia, D. S. Domingues, D. S. Sanches, and A. C. P. L. F. de Carvalho, “MathFeature: feature extraction package for DNA, RNA and protein sequences based on mathematical descriptors,” *Brief. Bioinform.*, vol. 23, no. 1, p. bbab434, Jan. 2022, doi: 10.1093/bib/bbab434.

[9] T. Akiba, S. Sano, T. Yanase, T. Ohta, and M. Koyama, “Optuna: A Next-Generation Hyperparameter Optimization Framework,” in *Proc. 25th ACM SIGKDD Int. Conf. on Knowledge Discovery & Data Mining*, in KDD ’19. New York, NY, USA, pp. 2623–2631, 2019, doi: 10.1145/3292500.3330701.

[10] M. Necci, D. Piovesan, Z. Dosztányi, and S. C. E Tosatto, “MobiDB-lite: fast and highly specific consensus prediction of intrinsic disorder in proteins,” *Bioinformatics*, vol. 33, no. 9, pp. 1402-1404, May 2017, doi: 10.1093/bioinformatics/btx015.
